# Hormonal Regulation of the MHC Class I Gene in Thyroid Cells: Role of the Promoter “Tissue-Specific” Region

**DOI:** 10.3389/fendo.2021.749609

**Published:** 2021-12-06

**Authors:** Cesidio Giuliani, Sara Verrocchio, Fabio Verginelli, Ines Bucci, Antonino Grassadonia, Giorgio Napolitano

**Affiliations:** ^1^ Unit of Endocrinology, Department of Medicine and Sciences of Aging, University “G. d’Annunzio” of Chieti-Pescara, Chieti, Italy; ^2^ Centre for Advanced Studies and Technology (CAST), University “G. d’Annunzio” of Chieti-Pescara, Chieti, Italy; ^3^ Department of Pharmacy, University “G. d’Annunzio” of Chieti-Pescara, Chieti, Italy; ^4^ Department of Oral, Medical and Biotechnological Science, University “G. d’Annunzio” of Chieti-Pescara, Chieti, Italy

**Keywords:** major histocompatibility complex class I (MHC-1), NF-kB, AP-1, thyroid, c-jun, p65, hormonal regulation

## Abstract

In previous studies we have demonstrated that the expression of the Major Histocompatibility Complex (MHC) class I gene in thyrocytes is controlled by several hormones, growth factors, and drugs. These substances mainly act on two regions of the MHC class I promoter a “tissue-specific” region (−800 to −676 bp) and a “hormone/cytokines-sensitive” region (−500 to −68 bp). In a previous study, we have shown that the role of the “tissue-specific” region in the MHC class I gene expression is dominant compared to that of the “hormone/cytokines-sensitive” region. In the present report we further investigate the dominant role of the “tissue-specific” region evaluating the effect of thyroid stimulating hormone (TSH), methimazole (MMI), phenylmethimazole (C10), glucose and thymosin-α1. By performing experiments of electrophoretic mobility shift assays (EMSAs) we show that TSH, MMI and C10, which inhibit MHC class I expression, act on the “tissue-specific” region increasing the formation of a silencer complex. Glucose and thymosin-α1, which stimulate MHC class I expression, act decreasing the formation of this complex. We further show that the silencer complex is formed by two distinct members of the transcription factors families activator protein-1 (AP-1) and nuclear factor-kB (NF-kB), c-jun and p65, respectively. These observations are important in order to understand the regulation of MHC class I gene expression in thyroid cells and its involvement in the development of thyroid autoimmunity.

## Introduction

Several studies have demonstrated that Major histocompatibility complex (MHC) class I as well as class II overexpression on non-immune cells are important in the development of autoimmune diseases and thyroid autoimmunity (1–9). Thus, there is an increased expression of MHC class I and II molecules in pancreatic β islet cells of patients with type 1 diabetes mellitus, in muscle biopsies of patients with inflammatory myopathies, and in thyrocytes from patients with autoimmune thyroid diseases (3, 5, 7). MHC class I overexpression is an early feature of the experimental-induced thyroiditis and this suggests its pathogenetic role in the autoimmune process (10, 11). Furthermore, MHC class I expression is necessary for the induction of systemic lupus erythematosus (SLE) and type 1 diabetes mellitus in experimental models of these diseases (1, 2). Indeed, down-regulation or absence of MHC class I expression is considered the hallmark of tissue immune privilege (4, 12). Regarding the thyroid, previous studies have demonstrated that hormones and growth factors that regulate thyroid cell growth and function, such as thyroid stimulating hormone (TSH), hydrocortisone, insulin and insulin-like growth factor-I (IGF-I) decrease MHC class I expression in a coordinate and specific way (7, 13, 14). Of note is that MHC class I expression is also decreased by iodide, phorbol esters, transforming growth factor (TGF)-β1, and methimazole (MMI), whereas it is increased by α-and γ-interferons (IFNs), thymosin-α1, and high levels of glucose (15–21). The specific regulation of MHC class I gene by these substances is particularly intriguing since the level of expression of MHC class I molecules differs between tissues, with the highest expression in lymphoid tissues and lower expression in some tissues as endocrine glands, skeletal muscle, myocardium, or gastric mucosa (22, 23). We believe that the levels of MHC class I molecules are lower in tissues that have potential autoantigens. Regarding the thyroid, we have hypothesized that the decrease of MHC class I molecules on the surface of thyrocytes by several hormones and factors, may be an important mechanism to preserve thyroid self-tolerance and prevent autoimmune thyroid disease. Indeed, the same hormones and factors increase the transcription of thyroid specific genes that can act as potential autoantigens (7, 16, 17). These data assume a particular importance considering the possibility that innate immune activation can lead to an autoimmune response. Pivotal studies have demonstrated that thyroid cells have functional pattern recognition receptors (PRRs), such as toll-like receptors (TLRs) and retinoic acid-inducible gene (RIG)-like receptors, that respond to various pathogen-associated molecular patterns (PAMPs) or damage-associated molecular patterns (DAMPs) (8, 24). Therefore, it has been hypothesized that the presence of PAMPs or DAMPs into thyroid cells can trigger an innate immune response, and eventually make thyrocytes to behave as antigen-presenting cells and to initiate an autoimmune response (25, 26). Experiments conducted in FRTL-5 cells have shown that the release of genomic ds DNA by injury activate several genes involved in the immune response including the MHC class I gene (26). We think that this mechanism is also true for other tissues expressing normally low levels of MHC class I molecules. Indeed, in patients with type 1 diabetes MHC class I overexpression is associated with pancreatic islets infiltration by cytotoxic T lymphocytes specific for autoantigens (27). Moreover, some further studies suggest that MHC class I overexpression is the trigger of immune-mediated myopathies (28, 29).

Given these data, we believe it is noteworthy that we know the regulation of MHC class I expression in thyroid cells to better understand the pathogenesis of autoimmune thyroid diseases and to detect how hormones and drugs can modulate the gene expression.

**Figure 1:**
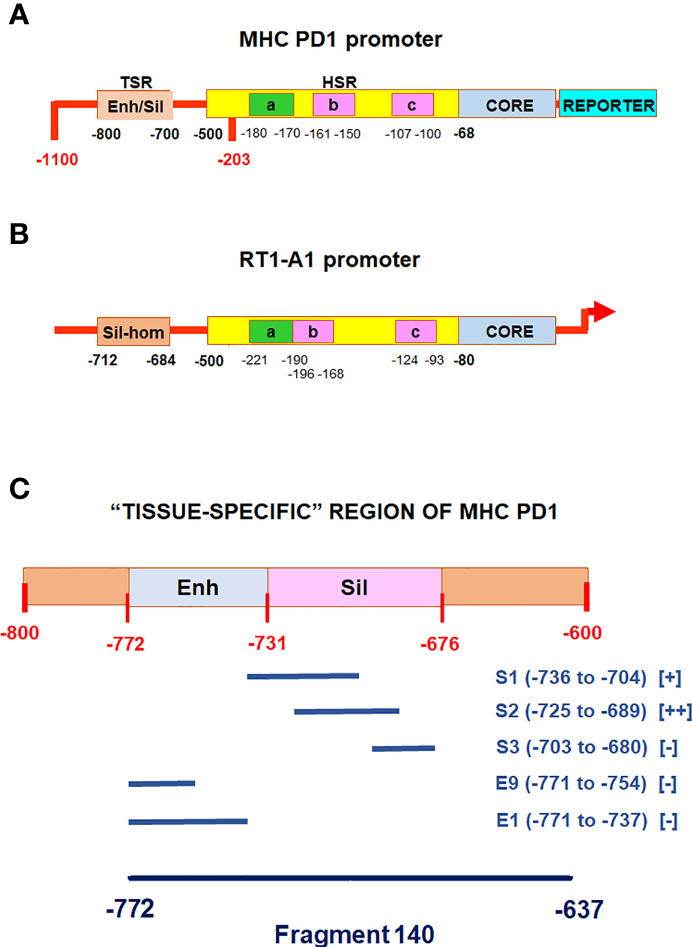
Diagrammatic representation of the MHC class I gene promoters. **(A)**, diagrammatic representation of the pig MHC class I PD1 promoter (6, 16). TSR: “tissue-Specific” Region including an overlapping enhancer and silencer (enh/sil) elements; HSR: “hormones/cytokines-sensitive” region including: the enhancer A (a), the interferon response element (b), the CRE-like element (c), the core promoter containing CAAT and TATAA-like elements (CORE); REPORTER: indicate the reporter gene used in previous studies for functional analysis by CAT or luciferase assays. **(B)**, diagrammatic representation of the rat MHC class I RT1-A1 gene promoter (Ensembl gene accession number: ENSRNOG00000038999). Sil-hom: a region homologous to the PD1 silencer of the “tissue-Specific” region; a: enhancer A, b: an overlapping interferon response element, c: enhancer B containing a CRE-like element, CORE: a region containing CAAT and TATAA elements; the arrow indicates the start of transcription. **(C)**, detailed diagram of the “tissue -specific” region of the MHC class I PD1 promoter, the relative positions of the oligonucleotides used as competitors in are indicated, as well as that of the 140 fragment used as labeled probe. [+] and [-] indicate presence or absence of competition, respectively.

Previous studies have defined the 5’ flanking region of the PD1 gene a swine classical MHC Class I gene whose properties are maintained when it is transfected into cells from different species (13–15, 17–20, 24, 30–32). It has got two main regions that control the expression of MHC class I gene in a particular cell **(** **Figure 1** **)**. The “tissue-specific” region, with overlapping enhancer and silencer elements, is -800 to -676 bp from the start of transcription; it sets the constitutive level of Class I expression in each tissue. The “hormone/cytokines-sensitive” region,” -500 to -68 bp, is responsible for the regulation of class I expression within each tissue and is modulated by the hormones, growth factors, drugs and cytokines mentioned above (6, 16). **Table 1** shows the effects of different hormones, growth factors, cytokines and drugs on the MHC class I promoter activity in the thyroid cell line FRTL-5, as described in previous studies (13–15, 17–20, 30). For all these compounds, except insulin and IGF-1, there is a similar effect both in the full length promoter -1100 bp from the start of transcription and in the deleted mutant -203 bp from the start of transcription, which lacks the “tissue-specific” region but retains the “hormone/cytokines-sensitive” region (see **Figure 1A** **)**. Experiments performed with reporter vectors containing mutations of one of the responsive elements of the “hormone/cytokines-sensitive” region [the enhancer A, the Interferon response element or the cyclic-AMP response (CRE)-like element], showed a loss of the effect of the related compounds and allowed the identification of this region (13–15, 17–20, 30). Furthermore, the disparate response of insulin and IGF-1 between the full length promoter and the deleted mutant p(-203) bp, have shown a dominant role of the upstream “tissue-specific” region in regulating the MHC class I gene transcription (30). The functional relationship between these two regulatory regions has been confirmed by the observation that insulin and IGF-1 lose their inhibitory effect on the activity of a full length MHC class I promoter that lacks the enhancer A element. Furthermore, electrophoretic mobility shift assays (EMSAs) have shown that both the “tissue-specific” region and the “hormone/cytokines-sensitive” region (particularly the enhancer A and the interferon response elements) interact with different members of the same family of transcription factors, nuclear factor-kB (NF-kB) and activator protein-1 (AP-1) (13–20, 30). In detail, the silencer element of the “tissue-specific” region interacts with the p65 subunit of NF-kB and c-jun, whereas the enhancer A element binds a protein complex named Mod-1 consisting of the p50 subunit of NF-kB and fra-2.

**Table 1:** Effects of the indicated compounds on the MHC class I promoter activity.

Compound	p (-1100)	p (-203)	Reference
TSH	Decrease	Decrease	(13)
Hydrocortisone	Decrease	Decrease	(14)
Insulin/IGF-1	Decrease	Increase	(30)
α-and γ-Interferons	Increase	Increase	(14, 18)
Iodide	Decrease	Decrease	(15)
TGF-β1	Decrease	Decrease	(19)
Thymosin-α1	Increase	Increase	(20)
Glucose	Increase	Increase	(18)
Methimazole	Decrease	Decrease	(17)
Phenylmethimazole	Decrease	Decrease	(17)

p(-1100) and p(-203) indicate a reporter vector containing respectively the full length (-1100 bp) or the deleted mutant (-203 bp) of the MHC class I promoter PD1. Data are from previous studies (13–15, 17–20, 30).

As shown in **Table 1**, the MHC class I gene expression is decreased by TSH, MMI and C10, whereas it is increased by glucose and thymosin-α1. These compounds act on the “hormone/cytokines-sensitive” region of the MHC class I promoter. In more detail, TSH, MMI and C10 induce the formation of specific protein/DNA complexes with a silencer sequence between -127 and -90 bp containing a CRE-like site (13, 17), whereas glucose and thymosin-α1 induce the formation of a protein/DNA complex, named Mod-1, with the Enhancer A sequence between -180 and -170 bp (18, 20). Since we have previously observed that the upstream “tissue-specific” region has got a dominant role in the regulation of MHC class I gene expression by insulin and IGF-1 (30), we have planned to evaluate the effects of TSH, MMI, C10, glucose and thymosin-α1 on this region.

In the present manuscript we illustrate that TSH, MMI, C10, glucose and thymosin-α1 act on the “tissue-specific” region of the MHC class I promoter modifying the formation of the silencer complex made up of two distinct members of the transcription factors families AP-1 and NF-kB, c-jun and p65 respectively. The effects of the aforementioned compounds on the silencer complex formation is consistent with those produced on the MHC class I gene expression. This observation further suggests that in the FRTL-5 cells the “tissue-specific” region acts as a dominant regulatory element of MHC class I promoter.

## Materials and Methods

### Materials

C10 was a gift from Intherthyr Research Corporation (Marietta, OH, USA) (17). Thymosin-α1 was kindly provided by SciClone Pharmaceuticals Inc (Foster City, CA, USA). [γ-^32^P]ATP (3000 Ci/mmol) and [α-^32^P]-dCTP (3000 Ci/mmol) were from Perkin Elmer Italia (Monza, Italy). Heat-treated, mycoplasma-free calf serum was from Life Technologies Europe (Monza, Italy). For EMSAs we used antibodies against the p65 (sc-8008) and p50 (sc-8414) subunits of NF-kB, c-jun (sc74543), and normal mouse IgG (sc-2025) as negative control (Santa Cruz Biotechnology Inc., Santa Cruz, CA, USA). The oligonucleotides S1, S2, S3, E1 and E9 were kindly provided by Dr. Dinah S. Singer (National Cancer Institute, NIH, Bethesda, MD, USA). Chromatin immunoprecipitation was carried out by using the following antibodies: NF-kB p65 (L8F6) mouse mAb, NF-kB p105/50 (D4P4D) rabbit mAb, c-Jun (60A8) rabbit mAb and normal rabbit or mouse IgG as negative control (Cell Signaling Technology Inc., Danvers, MA, USA). The source of all other materials was the Sigma Aldrich Co. (St. Louis, MO, USA), unless otherwise specified.

### Cell Culture

The F1 subclone of FRTL-5 rat thyroid cells (American Type Culture Collection, CRL-8305) was a gift from the Interthyr Research Foundation (Marietta, OH, USA). Cells were grown in 6H5% medium consisting of Coon’s modified Ham’s F-12 medium supplemented with 5% calf serum, 2 mM glutamine, 1 mM nonessential amino acids, and a six-hormone (6H) mixture: 1 mU/mL bovine TSH, 10 μg/mL insulin, 0.4 ng/mL cortisol, 5 mg/mL transferrin, 10 ng/mL glycyl-L-histidyl-L-lysine acetate, and 10 ng/mL somatostatin. These cells were diploid, between the 5^th^ and 25^th^ passage, and had all of the functional properties described previously (13–15, 17–21, 24, 30, 33–38). Fresh 6H5% medium was added to the cells every 2 to 3 days, and they were passaged every 7 days. In individual experiments, cells were grown to 60% confluence in 6H5% medium and then shifted to the appropriate treatment condition as previously reported (17, 18, 30). For the insulin treatment, cells were shifted to a three-hormone (3H) medium (i.e., with no TSH, insulin, or hydrocortisone) with only 0.2% calf serum (3H0.2%) ± insulin 10 μg/ml for 7 days. For the treatment with MMI, C10 and TSH, cells were shifted to a five-hormone (5H) medium (i.e., without TSH) with 5% calf serum (5H5% medium) for 7 days to become quiescent, and then treated with MMI 5 mM, C10 0.5 mM, or TSH 0.1 nM for 36 hours. In the experiments performed with thymosin-α1 and glucose, cells were shifted to 5H5% medium for 7 days to become quiescent, then they were cultured in 6H5% medium ± thymosin-α1 1 μg/mL for 12 hours, or 6H0.2% medium ± glucose 30 mM for 48 hours. The latter low serum medium was chosen to avoid a potential effect by variable concentrations of the glucose contained in the calf serum.

### Cell Extracts

Cellular extracts were prepared by a modification of described methods (39). In brief, cells were washed twice in cold PBS, pH 7.4, scraped, and centrifuged (500xg). The cell pellet was resuspended in 2 volumes of Dignam buffer C [25% glycerol, 20 mM 4-(2-hydroxyethyl)-1-piperazineethanesulfonic acid (HEPES-KOH), pH 7.9, 1.5 mM MgCl2, 0.42 M NaCl, 0.5 mM dithiothreitol, 1 μg/ml leupeptin, 1 μg/ml pepstatin and 0.5 mM phenylmethylsulfonyl fluoride]. The final NaCl concentration was adjusted based on cell pellet volume to 0.42 M. Cells were lysed by repeated cycles of freezing and thawing. The extracts were centrifuged (100,000xg) at 4°C for 20 minutes. The supernatant was recovered, aliquoted and stored at –70°C.

### EMSAs

The generation and preparation of the -140 bp probe and of the S1, S2, S3, E1 and E9 oligonucleotides were previously described (30, 31). Their sequences are reported in **Table 2**. The -140 bp probe was labeled with [γ-^32^P]ATP using T4 polynucleotide kinase (New England Biolabs, Ipswich, MA, USA), then purified on an 8% native polyacrylamide gel. EMSAs were performed as previously described (14, 30). Binding reactions in low salts and no detergent included 1.5 fmol [^32^P]DNA, 3 μg cell extract and 3 μg poly(dI-dC) in 10 mM Tris-Cl, pH 7.9, 1 mM MgCl2, 1 mM dithiothreitol (DTT), 1 mM ethylendiaminotetraacetic acid (EDTA), and 5% glycerol in a 20 μl total volume. Incubations were performed at room temperature for 30 min. Where indicated unlabeled oligonucleotides or antibodies were added to the binding reaction and incubated with the extracts for 20 minutes prior the addition of labeled DNA. Following incubations, reaction mixtures were electrophoresed on 4.5% native polyacrylamide gels at 160 V in 0.5x TBE at room temperature. Gels were dried and autoradiographed. Binding activity was quantified by optical densitometry using a STORM 860 Imager (Molecular Dynamics, GE Healthcare Italy, Milan, Italy) and data are shown in **Table 3**.

**Table 2:** Sequences (5’-3’) of the MHC class I PD1 promoter 140 fragment and of oligonucleotides S1, S2, E1 and E9 used as competitors in the EMSAs.

**140 fragment (-772 -637)**
GGTCCACATTCAAAATAACCTTTGAGAAATTACCATAATGATAGCATCCAAAATTATCTGAAAAGGTTATTAAAAATACATGTCCTACATGTGTGCGGGGCTTTTACATTTCATAGATGTCAGCCACCAAAAGGAG
**S1 oligonucleotide (-740 -700)**
C *GCG* AATGATAGCATCCAAAATTATCTGAAAAGGTTA *GCGC*
**S2 oligonucleotide (-727 -687)**
*GG* CCAAAATTATCTGAAAAGGTTATTAAAAATACATGTC *GG*
**E1 oligonucleotide (-772 -733)**
GGTCCACATTCAAAATAACCTTTGAGAAATTACCAT *CGC* G
**E9 oligonucleotide (-772 -746)**
GGTCCACATTCAAAATAAC*AGGA*G *C* G *C*

In blue the region -772 -732 spanning the enhancer element, in red the region -731 -676 spanning the silencer element. In italic and underlined are mutated nucleotides. See also ref. 31.

**Table 3:** Densitometric analysis of the EMSAs shown in **Figure 2A** and **Figures 3A, B, C**.

Treatment	Band intensity (arbitrary unit)
Control 3H0.2%	100
+ Insulin	289 ± 7.2*
Control 5H5%	100
+ MMI	267 ± 8.4*
+ C10	269 ± 6.6*
+ TSH	233 ± 9.1*
Control 6H5%	100
+ Thymosin-α1	58 ± 5.0*
Control 6H0.2%	100
+ Glucose	44 ± 6.9*

The intensity of each relative control is set to 100. Data are means ± S.D from three independent experiments. *p < 0.05 compared to relevant control.

### RNA Isolation and Northern Analysis

RNA was prepared using a RNeasy Mini kits (Qiagen Inc., Valencia, CA, USA). Twenty μg of the different RNA samples were run on denaturing agarose gels, capillary blotted on Nytran membranes (Schleicher & Schuell-Whatman, Florrham Park, NJ, USA), UV cross-linked, and hybridized by using QuickHyb Hybridization Solution (Stratagene, La Jolla, CA, USA), following the manufacturer protocol. The probes were labeled with [α-32P]-dCTP using Ladderman Labeling kits (Takara Mirus Bio, Madison, WI, USA). The MHC class I probe was a 1.0 kb HpaI fragment of the MHC class-I pH 7 clone spanning the entire cDNA insert (40). The β-actin probe was as described previously (40). Quantitation was performed using the STORM 860 Imager (Molecular Dynamics).

### Chromatin Immunoprecipitation (ChIP) Assay

ChIP was performed using SimpleChIP enzymatic chromatin IP kit (Cell Signaling Technology Inc.) following the manufacturer instructions. In brief, FRTL-5 cells cultured in 3H0.2% ± insulin 10 μg/ml for 7 days, as described above, were crosslinked for 10 min at room temperature by 1% formaldehyde. Nuclear DNA was digested by Micrococcal nuclease and nuclei were lysed by ultrasonic homogenizer. The complexes were purified by ChIP grade protein G agarose and the crosslinks were reversed incubating the samples with 200 mM NaCl and 120 μg of Proteinase K for 2 hours at 65°C. Chromatin immunoprecipitation was performed with the antibodies indicated above, DNA was purified and analyzed using real-time quantitative PCR (qPCR).

### qPCR Analysis

qPCR was performed using SimpleChIP Universal qPCR master mix (Cell Signaling Technology Inc.) and a primer pair (forward: 5’-TCAAGGCCAGCTTGGTCTAC-3’; reverse: 5’-CAGCAGCCCAGCAGCCTC-3’) flanking a region of the rat MHC class I gene RT1.A1 (Ensembl gene accession number: ENSRNOG00000038999) homologous to the silencer element of the “tissue-specific” region of the PD1 promoter used in the EMSAs. Each sample was run, in triplicate, in a QuantStudio 7 PRO Real-Time PCR System (Applied Biosystem, ThermoFisher Scientific, Waltham, MA, USA) and the immunoprecipitation efficiency was calculated using the percent input method.

### Other Assays

Protein concentrations were determined using BCA protein assay kits (Pierce Biotechnology Inc., Rockford, IL, USA), with crystalline BSA as standard.

### Statistical Analysis

All experiments were repeated at least three times with independent batches of cells. The quantitative data obtained by optical densitometry were evaluated as means ± S.D. The significance between experimental values was determined by unpaired two-tailed t-test or two-way ANOVA, with p < 0.05 or better when the data from all of the experiments were considered.

## Results

### EMSAs Indicate That the Functional Effects of TSH, Glucose, Thymosin α-1, MMI and C10 on MHC Class I Gene Expression Correlate With Changes in Binding of DNA/Protein Complexes to the “Tissue-Specific” Region of the PD1 Promoter

**Figure 2:**
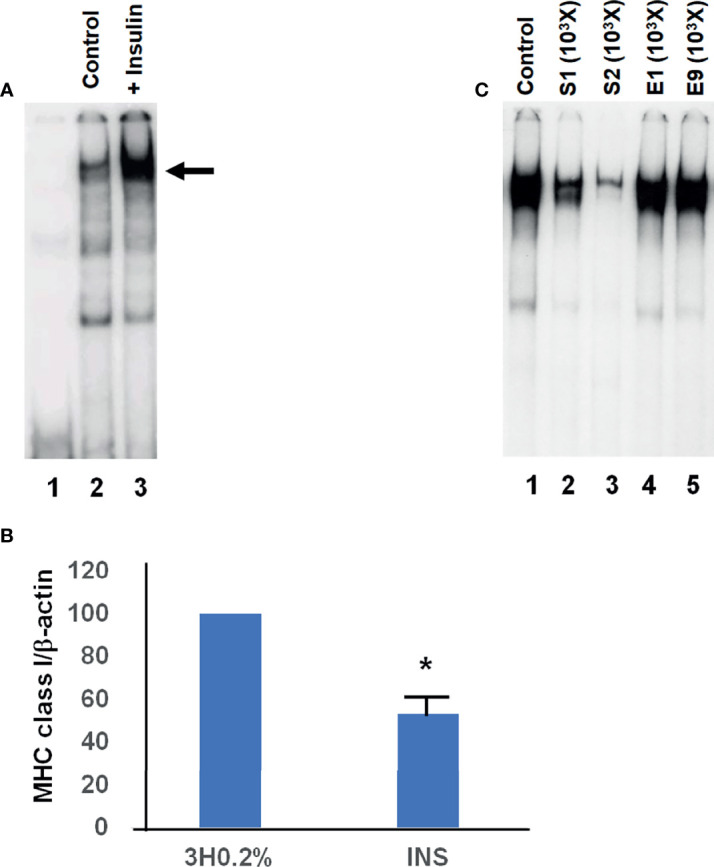
Representative EMSAs showing the effect of insulin on the formation of the silencer element. **(A)**, EMSAs were performed, as detailed in Material and Methods, using cellular extracts from FRTL-5 and the –140 bp probe that spans the region between –772 to –637 bp from the transcription start site (this fragment encloses the overlapping enhancer and silencer elements of the “tissue-specific” region). Lane 1, radioactive probe alone; lane 2 is the incubation of radioactive probe with extracts from control cells (3H 0.2% medium); lane 3 is the incubation of radioactive probe with extracts from cells in 3H 0.2% medium + insulin 10 μg/ml. The arrow indicates the protein-DNA complex identified as the silencer element. **(B)**, effect of insulin treatment on MHC class I RNA levels. FRTL-5 cells were grown and treated as in the EMSAs shown in (A). Data are means MHC class-I/β-actin ± S.D. expressed as percentages of control from three separate experiments, *p < 0.05 compared to control. INS, cells in 3H 0.2% medium + insulin 10 μg/ml. **(C)**, EMSAs were performed, as detailed in Material and Methods, using cellular extracts from FRTL-5 and the –140 bp probe that spans the region between –772 to –637 bp from the transcription start site. Lane 1 is the incubation of radioactive probe with extracts from cells maintained in 3H 0.2% medium + insulin 10 μg/ml; lanes 2 to 5 show the effect of the unlabeled oligonucleotides S1, S2, E1 and E9 (1000 fold excess) on the complex formation. Data are from a representative experiment repeated three times with similar results.

We have performed EMSA experiments using a radiolabeled probe with a sequence encompassing the MHC class I promoter between -772 to -637 bp, termed 140 fragment because of its total length (including nucleotides from restriction sites on either end). This fragment encloses the overlapping enhancer and silencer elements of the “tissue-specific” region previously described (31, 32). As previously showed (30), the addition of insulin 10 μg/ml to the FRTL-5 cells maintained in a 3H medium culture (i.e., no TSH, insulin, or hydrocortisone and containing only 0.2% calf serum) increased the formation of a slowing complex **(** **Figure 2A** lane 3, arrow; **Table 3** **)** similar to a previous identified silencer of the promoter transcription (31, 32). This treatment decreased the MHC class I RNA level **(** **Figure 2B** **)** as already observed in previous studies (41).

**Figure 3:**
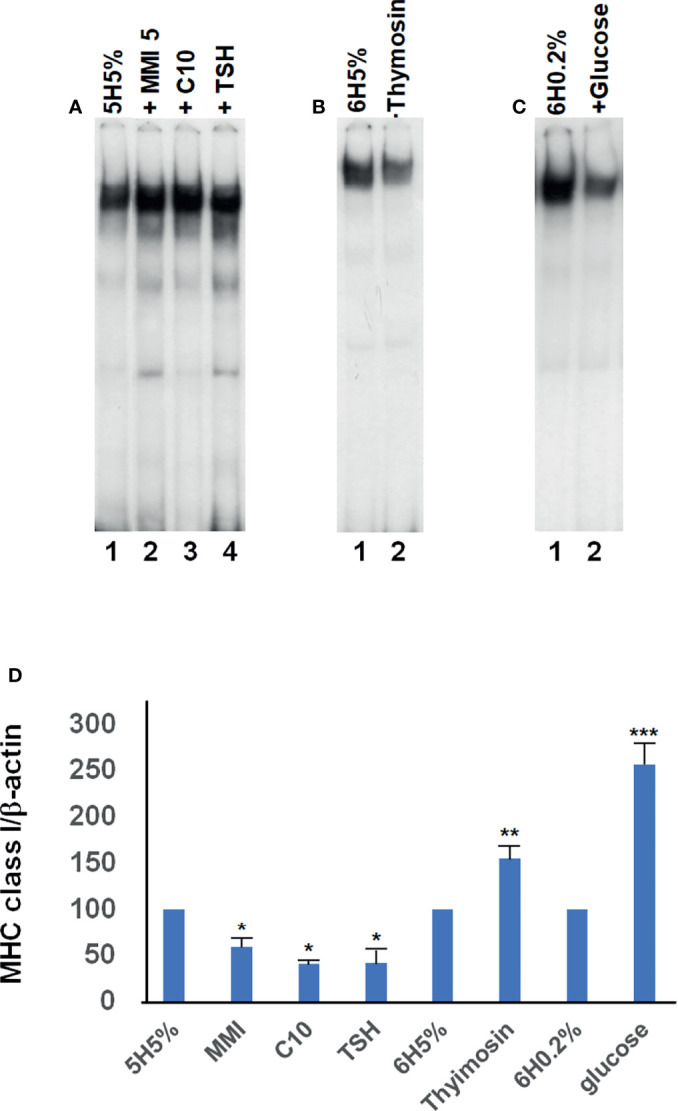
Representative EMSAs showing the effect of different compounds on the formation of the silencer element. EMSAs were performed, as detailed in Material and Methods, using cellular extracts from FRTL-5 and the –140 bp probe that spans the region between –772 to –637 bp from the transcription start site (this fragment encloses the overlapping enhancer and silencer elements of the “tissue-specific” region). **(A)**, Lane 1 is the incubation of the radioactive probe with extracts from control cells (5H 5% medium, i.e. without TSH); lanes 2 to 4 show the effect of MMI 5 mM, C10 0.5 mM, and TSH 0.1 nM, respectively, on the complex formation after 36 hours of treatment. **(B)**, lane 1 is the incubation of radioactive probe with extracts from control cells (6H 5% medium); lanes 2 show the effect of thymosin-α1 1 μg/mL on the complex formation after 12 hours of treatment. **(C)**, lane 1 is the incubation of radioactive probe with extracts from control cells (6H 0.2% medium); lanes 2 show the effect of glucose 30 mM on the complex formation after 48 hours of treatment. Data are from a representative experiment repeated three times with similar results. Differences are statistically significant, p < 0.05. **(D)**, effect of the treatments performed in EMSAs on the MHC class I RNA levels. FRTL-5 cells were grown and prepared as detailed in Materials and Methods. Data are means MHC class-I/β-actin ± S.D. expressed as percentages of control from three separate experiments, *p < 0.05 compared to the relative control 5H5%; **p < 0.05 compared to the relative control 6H5%; ***p < 0.05 compared to the relative control 6H0.2%.

The identification of this complex with the silencer element previously described (30, 31) was confirmed by competition experiments. Indeed, the preincubation of the cellular extracts with unlabeled double-strand oligonucleotides, spanning the functional silencer element previously identified and named S1 and S2 (30, 31) **(** **Figure 1C** **)** inhibited its formation **(** **Figure 2C** lanes 2 and 3 *vs*. 1, respectively). This inhibition was specific since two oligonucleotides, corresponding to the enhancer portion of the “tissue-specific” regions **(** **Figure 1C** **)**, named E1 and E9 (31) did not decrease the complex **(** **Figure 2C** lanes 4 and 5 *vs*. 1, respectively). It must be emphasized that high levels of this silencer complex are associated with low levels of MHC class I expression (6, 30–32). EMSAs experiments performed by using extracts from cells treated with TSH, MMI, C10, glucose and thymosin-α1, confirmed the consistency between the silencer formation and the expression of the MHC class I gene **(** **Figure 3** **)**. Indeed, cellular extracts from cells cultured in 5H medium (i.e. without TSH) and 5% calf serum treated with compounds that cause a decrease of MHC class I expression, as MMI 5 mM, or C10 0.5 mM, or TSH 0.1 nM, showed an increase of the silencer complex **(** **Figure 3A** lanes 2, 3, 4 *vs*. 1, respectively; **Table 3** **)**. Conversely, cellular extracts from cells treated with compounds that cause an increase of MHC class I expression, as thymosin-α1 1 μg/mL or glucose 30 mM, showed a decrease of the silencer complex **(** **Figure 3B** lane 2 *vs*. 1 and **Figure 3C** lane 2 *vs*. 1, respectively; **Table 3** **)**. The choice of treatment conditions is based on our previous experiments (17, 18, 20) and are detailed in the *Materials and Methods* section. **Figure 3D** shows the effect of these treatments on the MHC class I RNA level, which confirm our previous studies

**Figure 4:**
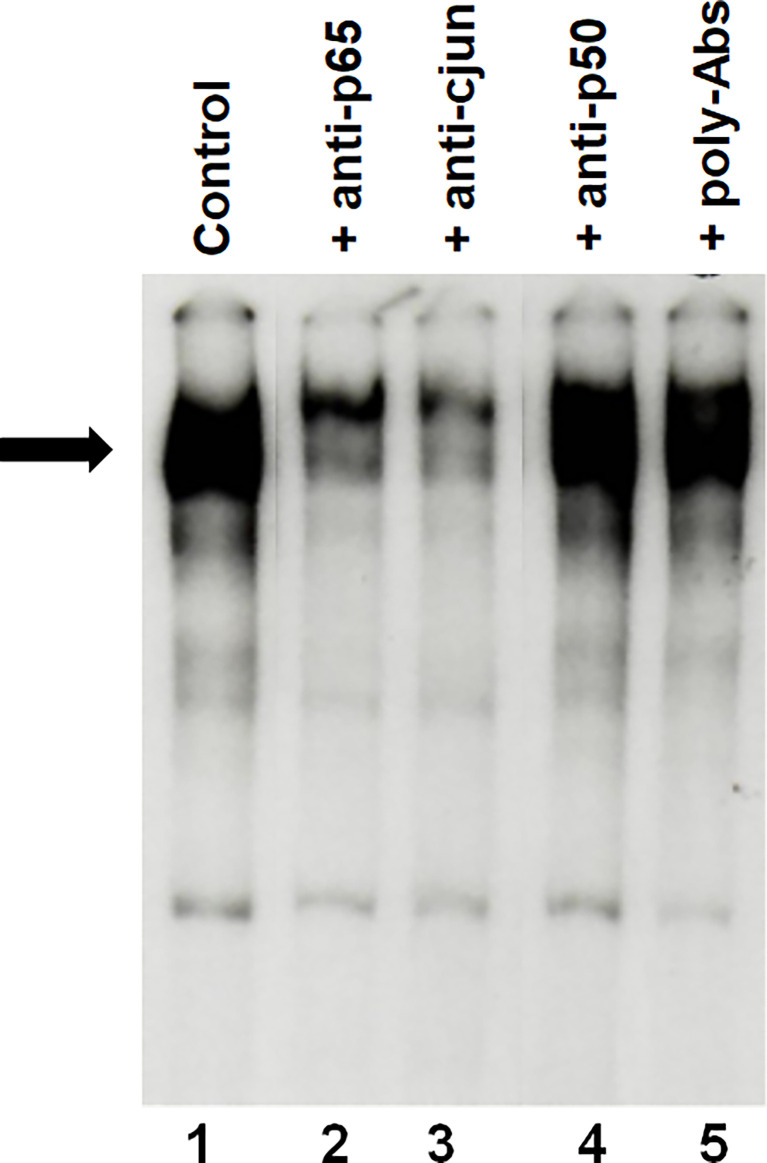
Representative EMSAs showing the ability of antisera specific for the p65 subunit of NF-kB or c-jun to inhibit the formation of the silencer complex. EMSAs were performed, as detailed in Material and Methods, using cellular extracts from FRTL-5 and the –140 bp probe that spans the region between –772 to –637 bp from the transcription start site. Lane 1 is the incubation of the radioactive probe with extracts from control cells (6H 5%); lanes 2 to 6 are the incubation of the radioactive probe with extracts from control cells after preincubation with antibodies directed against the p65 subunits of NF-kB, c-jun and the p50 subunit of NF-kB respectively; lane 5 refers to preincubation with normal polyclonal mouse IgGs as negative control. Data are from a representative experiment repeated three times with similar results. Differences are statistically significant, p < 0.05.

Cell extracts from FRTL-5 cells maintained in 6H5% medium were then preincubated with antibodies against the p65 subunit of NF-kB and c-jun. This experiment has shown a decrease of the silencer complex **(** **Figure 4**, lanes 2 and 3 respectively), whereas no effect was seen using antibodies against the p50 subunit of NF-kB **(** **Figure 4**, lane 4 *vs*. 1). This decrease indicates an involvement of c-jun and p65 in the formation of the silencer complex as previously observed using extracts from FRTL-5 cells treated with IGF-1 or insulin (30).

### ChIP Assay Confirms the Binding of c-jun and the p65 Subunit of NF-kB to the “Tissue-Specific” Region of the Rat MHC Class I Promoter

**Figure 5:**
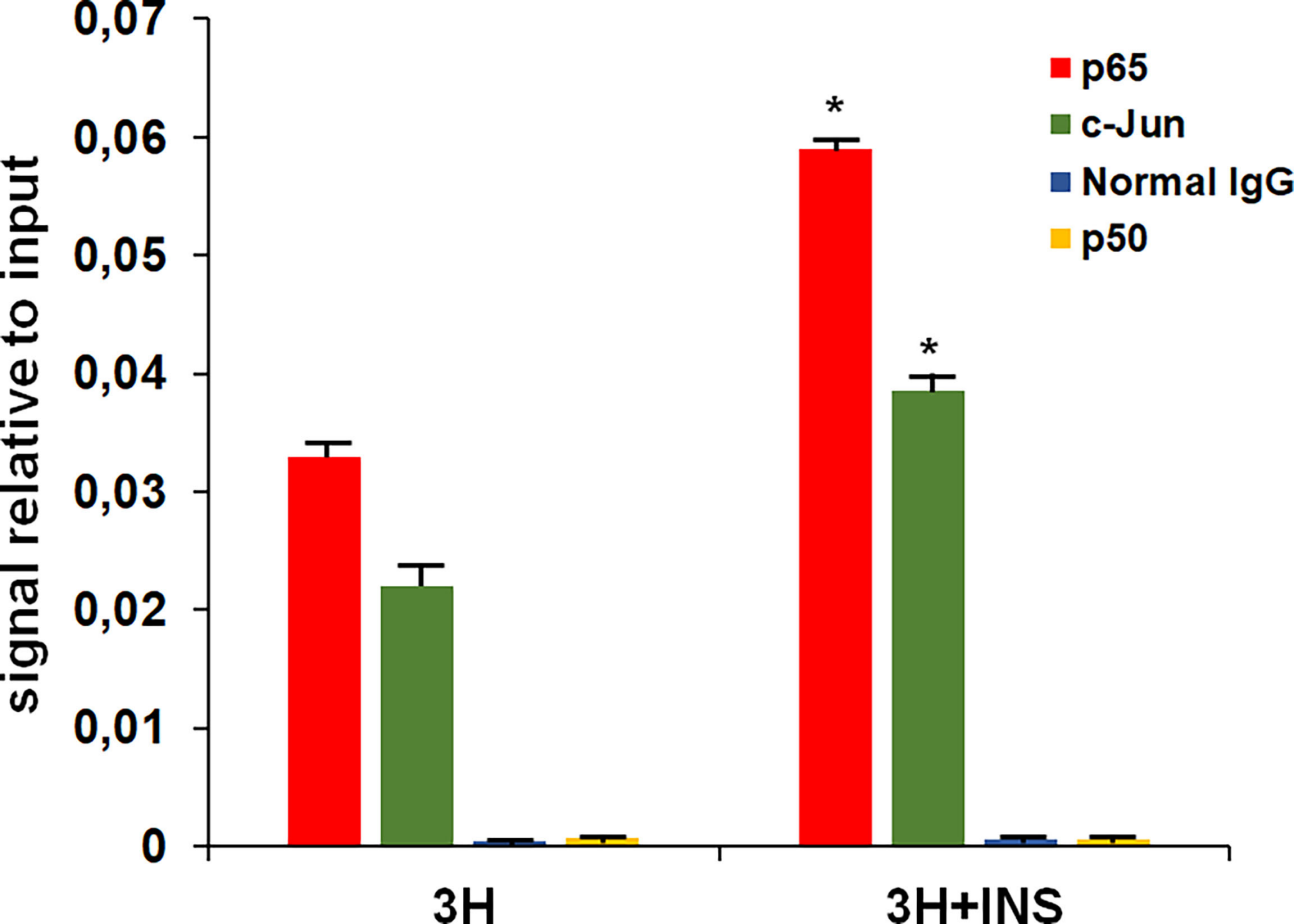
qPCR analysis of purified DNA from ChIP performed using antibodies against the p65 subunit of NF-kB and c-jun. 3H, control cells (3H 0.2% medium); 3H + INS (3H 0.2% medium + insulin 10 μg/ml). Normal rabbit or mouse IgG were used as negative control in the ChIP assay. The amount of immunoprecipitated DNA in each sample is represented as signal relative to the total amount of input chromatin. Data are means ± SD for three independent experiments. *p < 0.05 *versus* relevant control.

EMSAs, although very useful for analyzing the interaction between transcription factors and specific DNA sequences, it has the limit of carrying out the reaction in a test tube. Therefore, we have evaluated *in vivo* using ChIP assay the interaction between c-jun and the p65 subunit of NF-kB with a region of the promoter of the rat MHC class I (RT1-A1 gene) homologous to the silencer element of the “tissue-specific” region of the MHC class I PD1 promoter. The cross-linked chromatin complex was obtained from FRTL-5 cells treated with 3H 0.2% ± insulin 10 μg/ml for 7 days and immunoprecipitation was performed using antibodies against the rat c-jun and the rat p65 subunit of NF-kB. The purified DNA was then analyzed by qPCR using primers flanking the rat homologous silencer element. As shown in **Figure 5** the experiment confirmed the binding of the transcription factors c-jun and the p65 subunit of NF-kB with this region and the treatment with insulin increased the protein-DNA interaction as observed with EMSAs. No effect was seen in using antibodies against the rat p50 subunit of NF-kB.

## Discussion

Several studies performed in the past have demonstrated the effects of various hormones, growth factors, cytokines and drugs on MHC class I gene expression. They have also shown that these compounds act on a region between -500 to -68 bp, therefore called “hormone/cytokines-sensitive” region. This region has got several enhancer and silencer elements as illustrated in **Figure 1**. In more details, those studies have shown that some compounds such as TSH, MMI and C10 act inducing the formation of two protein–DNA complexes on a region that includes the constitutive 38 bp silencer of the MHC class-I promoter, which contains a CRE-like sequence (11, 15). Other compounds as hydrocortisone, insulin/IGF-1, TGF-β1, thym and glucose act regulating the binding of the Mod-1 complex with the enhancer A element (12, 16–18, 22). In most cases, an increase in Mod-1 binding stimulates the gene transcription, while a decrease inhibits it. However, we have previously observed (22) that although insulin or IGF-1 makes Mod-1 binding increase it leads the transcription of the MHC class I gene to be reduced. This discrepancy was explained by the observation that insulin and IGF-1 also increased the binding of a protein complex to a silencer element located upstream on the promoter in the “tissue-specific” region. From these results, which have highlighted a dominant role of this region in the MHC class I gene regulation, we were prompted to evaluate the effects of other factors on it.

As a first attempt we have chosen to evaluate the effect of either compounds that decrease the MHC class I gene expression, such as TSH, MMI and C10, or compounds that increase it, such as thymosin-α1 and glucose. TSH was chosen since it is the main regulator of thyroid growth and function. MMI is a drug widely used to treat hyperthyroidism that, beside an antithyroid effect, also has anti-inflammatory and immunosuppressive effects (42–44). C10, a phenyl derivative of MMI (17), is a more potent anti-inflammatory agent both *in vitro* and *in vivo* (17, 45). Thymosin-α1 is a drug used to stimulate the immune response (46) and one of its mechanisms of action is the increase of MHC class I expression (20, 47). The increased expression of MHC class I molecules on thyroid cells induced by the high glucose levels can be involved in the higher incidence of thyroid autoimmunity associated with hyperglycemia (48, 49). Furthermore, an increased expression of MHC class I molecules in endothelial cells has been associated with coronary artery disease (50) and this can be related with the vascular complications induced by hyperglycemia.

In the present study we have demonstrated that in addition to insulin and IGF-1, even TSH, MMI, C10, thymosin-α1 and glucose regulate the silencer complex in the “tissue-specific” region and their effects on this site are in tune with the effects they have on the downstream “hormone/cytokines-sensitive” region. These data further suggest that in FRTL-5 cells the “tissue-specific” region acts as a dominant regulatory element. They also show that for most of the compounds tested the two regulatory regions of the MHC class I promoter act in concert with each other and the only exception observed so far is for insulin and IGF-1. The functional relationship between the “tissue-specific” region and the “hormone/cytokines-sensitive” region has been observed in a previous study where we evaluated the effect of insulin and IGF-1 on a reporter vector containing the full length of the MHC class I promoter with a deletion of the enhancer A element (30). The deletion of the enhancer A site resulted in the loss of the insulin/IGF-1 effect on the promoter activity. A weakness of the present work is the lack of information on the effects of other compounds involved in the autoimmune process. Further studies need to be performed in order to find whether other compounds, particularly cytokines, have a disparate response between these two regulatory regions. Moreover, further studies should evaluate the regulation of MHC class I by hormones, growth factors and cytokines in other tissues beside thyroid.

In the present study, we have also noticed that in FRTL-5 cells the transcription factors involved in the upstream silencer complex formation are the p65 subunit of NF-kB and the AP-1 family member c-jun. It must be underlined that the two primary regions that regulate MHC class I transcription in thyroid cells, that are the “tissue-specific” region and the “hormone/cytokines-sensitive” region, interact with different members of the same family of transcription factors. Indeed, the upstream silencer interact with the p65 subunit of NF-kB and c-jun, whereas the downstream enhancer A element binds either the complex Mod-1 (formed by the heterodimer p50/fra-2) or the classic NF-kB dimer p50/p65, which has an opposite effect compared to Mod-1 (14, 15). Thus, it is possible that a regulatory effect in one subunit can affect the other and alter the way in which these factors regulate the promoter. Further studies are needed to deepen our knowledge on the functional interactions between these transcription factors and the promoter regions, as well as to evaluate if other transcription factors are involved.

Of particular interest is also the observation that MHC class I expression in thyroid cells is controlled by several hormones and growth factors. This hormonal regulation is considered important for the suppression of autoimmunity during hormonally induced changes in thyroid cell function, which results in an enhanced expression of potential thyroid autoantigens, such as thyroglobulin, thyroid peroxidase, and the TSH receptor (7, 8). Indeed, several observations suggest that the overexpression of HLA molecules on nonimmune cells has an important role in the pathogenesis of autoimmune diseases. It has been hypothesized that several insults to nonimmune cells of target tissues (such as viral infections, dsRNA, ds DNA or tissue injury) can activate the innate immune response through PRRs such as TLRs and RIG-like receptors, and induce the secretion of α-, β- or γ-IFN. This would result in an overexpression of MHC molecules and cytokines by the target cells, which would then recruit and activate lymphocytes and hence initiate an autoimmune response (7, 8, 24–26, 51). Therefore, we think it is very important to know of the mechanisms that regulate the MHC class I gene as well as the compounds that can modify them. Indeed, it is of remarkable importance to underline is the selective regulation of the distinct members of NF-kB and AP-1 by several hormones and growth factors. The hormonal regulation of the NF-kB dimers in thyroid cells is not restricted to the MHC gene. Studies performed in the FRTL-5 cells have shown that TSH can modify the composition of the NF-kB dimers activated by TNF-α. In the absence of TSH, TNF-α treatment activates only the p50 homodimers, whereas in the presence of TSH there is also the activation of the p50/p65 heterodimers, which results in the modification of the IL-6 gene expression (52).

The data presented herein suggest that the “tissue-specific” region represents the primary regulator of the MHC class I gene transcription in thyroid cells. They further suggest that some drugs such as MMI, its derivative C10 and thymosin-α1 regulate the binding of c-jun and the p65 subunit of NF-kB to this region. This can have an impact on the therapy of autoimmune and inflammatory diseases as well as on the treatment of cancer. Indeed, we speculate that the effectiveness of MMI in the treatment of autoimmune thyroid diseases is not only due to its antithyroid effect but also to its ability to suppress the inflammatory and immune processes. For this reason, we and other groups have studied MMI derivatives characterized by a high anti-inflammatory and immunosuppressive potency and a low or no effect on thyroid function (17, 45, 53, 54). C10 is one of these derivatives and several studies suggest its potential use in severe inflammatory diseases (45). On the other hand, the information obtained on the mechanism of action of thymosin-α1 are important for its use in cancer immunotherapy and to stimulate the research of new drugs with the same mechanism of action, considering that the loss of MHC class I expression is a feature of several tumors including papillary thyroid cancer (55–59).

The present study, as well as most of the studies discussed here, has been performed in the FRTL-5 cells. They are a non-transformed rat thyroid cell line in continuous culture that represents a well-defined and reproducible in-vitro model of thyroid function (13–15, 17–21, 24, 30, 33–38). The reliability of the FRTL-5 cells as a model to study MHC gene regulation has been validated by studies conducted in animal models and in human tissues (1, 7, 8, 54, 60, 61).

In summary, our data show that the “tissue-specific” region of the MHC class I promoter is the target of several hormones and growth factors that regulate the gene expression. Future research directions should be performed to deepen the knowledge about MHC class I promoter activity in thyroid and in other tissues, either nonimmune and immune and to understand its role in autoimmune diseases and cancer.

## Data Availability Statement

The raw data supporting the conclusions of this article will be made available by the authors, without undue reservation.

## Author Contributions

CG and GN designed and drafted the manuscript. The experimental procedures and data analysis were performed by CG, SV, FV, IB, AG, and GN. All authors contributed to the article and approved the submitted version.

## Conflict of Interest

The authors declare that the research was conducted in the absence of any commercial or financial relationships that could be construed as a potential conflict of interest.

## Publisher’s Note

All claims expressed in this article are solely those of the authors and do not necessarily represent those of their affiliated organizations, or those of the publisher, the editors and the reviewers. Any product that may be evaluated in this article, or claim that may be made by its manufacturer, is not guaranteed or endorsed by the publisher.
